# Enhanced and Secretory Expression of Human Granulocyte Colony Stimulating Factor by *Bacillus subtilis SCK6*

**DOI:** 10.1155/2015/636249

**Published:** 2015-12-31

**Authors:** Shaista Bashir, Saima Sadaf, Sajjad Ahmad, Muhammad Waheed Akhtar

**Affiliations:** ^1^School of Biological Sciences, University of the Punjab, Lahore 54590, Pakistan; ^2^Institute of Biochemistry and Biotechnology, University of the Punjab, Lahore 54590, Pakistan

## Abstract

This study describes a simplified approach for enhanced expression and secretion of a pharmaceutically important human cytokine, that is, granulocyte colony stimulating factor (GCSF), in the culture supernatant of* Bacillus subtilis *SCK6 cells. Codon optimized GCSF and pNWPH vector containing SpymwC signal sequence were amplified by prolonged overlap extension PCR to generate multimeric plasmid DNA, which was used directly to transform* B. subtilis *SCK6 supercompetent cells. Expression of* GCSF *was monitored in the culture supernatant for 120 hours. The highest expression, which corresponded to 17% of the total secretory protein, was observed at 72 hours of growth. Following ammonium sulphate precipitation, GCSF was purified to near homogeneity by fast protein liquid chromatography on a QFF anion exchange column. Circular dichroism spectroscopic analysis showed that the secondary structure contents of the purified GCSF are similar to the commercially available GCSF. Biological activity, as revealed by the regeneration of neutrophils in mice treated with ifosfamine, was also similar to the commercial preparation of GCSF. This, to our knowledge, is the first study that reports secretory expression of human GCSF in* B. subtilis *SCK6 with final recovery of up to 96 mg/L of the culture supernatant, without involvement of any chemical inducer.

## 1. Introduction

The development of efficient systems for the production of biosimilars is one of the key targets of the biotechnology industry.* Escherichia coli*, by far, is regarded as one of the convenient hosts for the recombinant production of therapeutically important and commercially relevant proteins [1–3]. However, overexpression of many recombinant proteins in this host leads to the accumulation of desired product in the form of inclusion bodies (IBs), which are biologically inactive. Whereas the additional steps required in the recovery of bioactive protein from the IBs result in an overall low yield, the presence of lipopolysaccharides (endotoxins) in the outer membrane of* E. coli* further complicates the purification scheme and hence limits the usefulness of this system ([4–7] and references therein).

Targeting expression of heterologous proteins in the culture medium may be an attractive choice as it may reduce the downstream processing cost [8]. In this regard, Gram-positive bacterium* Bacillus subtilis*, which directly exports proteins into the extracellular medium, may be exploited well [6, 9].* B. subtilis*, owing to its naturally high secretory ability, provides better folding conditions and thus prevents formation of IBs as opposed to the* E. coli* based expression systems [10, 11]. Its Sec-dependent secretary pathway is involved in the formation of secretory preproteins complex with the chaperons that bind to the secretory translocase and help in translocation across the cytoplasmic membrane. The protein is released from translocase after removal of signal peptide, refolded, and crosses the cell wall [8, 10, 12, 13]. Low protein yield, abundant secretion of proteases, and plasmid instability, however, are some bottlenecks which may sometime limit the application potential of* B. subtilis* ([9] and references therein).

Neutropenia, that is, decreased count of neutrophils, is one of the most common side effects of chemotherapy and/or bone marrow transplantation. Human granulocyte colony stimulating factor (GCSF) is an important biosimilar that plays important role in survival, proliferation, and activation of neutrophils and thus reduces morbidity rate in patients [14, 15]. It is amongst the few cytokines that have been used in clinical trials with diverse applications, that is, the stem cell mobilization, treatment of central nervous system disorders like cerebral ischemia and stroke, regeneration of hepatic tissues, and so forth [16–18]. Cloning and expression of this therapeutically important cytokine (~19 kDa protein) have been reported by several research groups in* E. coli* but in the form of IBs [14, 19, 20]. Achieving* GCSF* expression in native-like, biologically active form, however, is a more attractive option.

The present study was designed with an objective to generate a vector-host system that may be exploited for the cost-effective production of human GCSF in soluble and bioactive form.* B. subtilis* expression host, which is “generally regarded as safe” by the US Food and Drug Administration, has been utilized in combination with pNWPH vector that contains a strong promoter (P_HbaII_) and SpymwC signal sequence for improved secretion of GCSF into the culture medium. A simplified approach for simultaneous amplification of the vector and the insert DNAs followed by direct transformation of the multimeric recombinant DNA into the expression host is also described here. This, to our knowledge, is the first report that explains multimeric cloning, enhanced and secretory, cost-effective production of human GCSF in* B. subtilis* SCK6. The study is likely to contribute to developing biosimilars by the biopharmaceutical companies, for diverse applications and analysis.

## 2. Materials and Methods

### 2.1. Chemicals, Kits, Plasmids, and Bacterial Strains

All chemicals and kits used in the present study were of highest purity grade commercially available.* Pfu* DNA polymerase, dNTPs, DNA, and protein size markers were purchased from Thermo Scientific (USA). The designed oligonucleotides used in POE-PCR were acquired from Oligo Macrogen (USA).

Plasmid pNWPH and the* B. subtilis* SCK6 (http://www.bgsc.org/viewdetail.php?bgscid=1A976&Search=sck) bacterial strain, used in this study, were a kind gift from Dr. X.-Z. Zhang [21], Virginia Polytechnic Institute and State University, Blacksburg, VA 24061, USA. Media used for the growth of* B. subtilis* were Luria-Bertani [LB (1% tryptone, 0.5% yeast extract, 1% NaCl, and pH 7)] and the modified 2x L-Mal medium (2% tryptone, 1% yeast extract, 1% NaCl, 7.5% maltose hydrate, and 7.5 *μ*g/mL MnSO_4_). Chloramphenicol and erythromycin, at a final concentration of 5 and 1 *μ*g/mL, respectively, were used as selection antibiotics.

### 2.2. Recombinant Plasmid Construction

**Figure 1:**
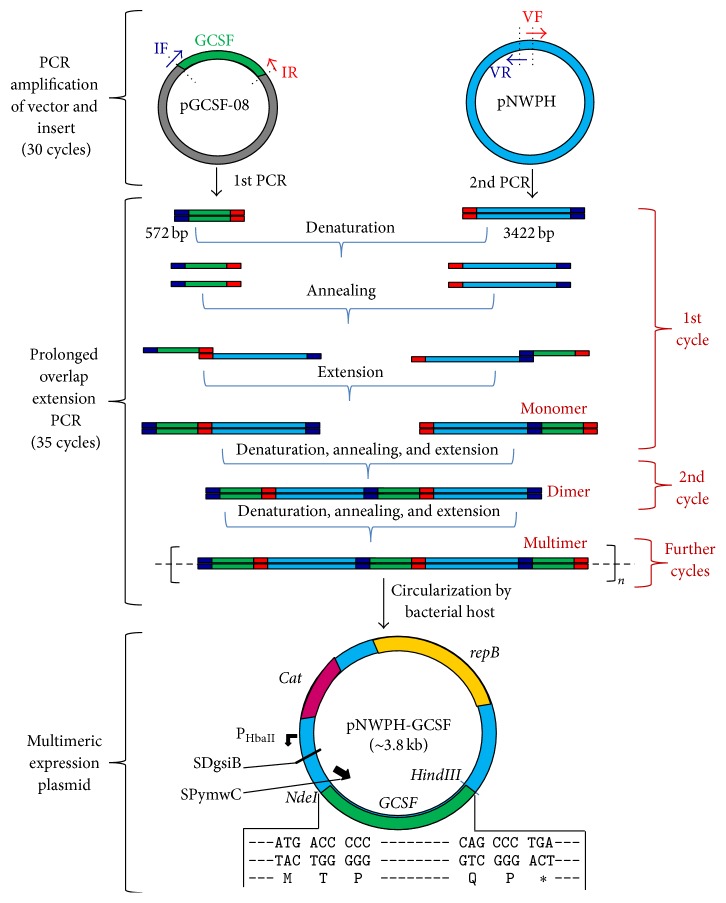
Construction of the pNWPH-GCSF expression plasmid using prolonged overlap extension PCR/multimeric cloning strategy. Simple PCR generated 3′ and 5′ overhangs of insert (GCSF) and vector (pNWPH). These overhangs acted as primers during the formation of multimers. Circular plasmid pNWPH-GCSF was thereafter generated in* B. subtilis* by direct transformation of multimers containing* GCSF* gene. repB, replication protein B; Cat, chloramphenicol transferase gene; P_HbaII_, promoter; SDgsiB, Shine-Dalgarno sequence of the gsiB gene; SPywmC, signal sequence.

Plasmid pNWPH-mini-scaf [22] containing chloramphenicol resistance gene, a strong P_HpaII_ promoter and SPymwC signal sequence, was used for the construction of pNWPH-GCSF (Figure 1). The primers used for the multimer cloning were comprised of 50 nucleotides (nt) each, having 25 nt overlapping region of the insert and 25 nt of the vector (Table 1). The codon optimized gene of human GCSF (*KT326155*) was amplified from pGCSF-08 construct of our lab (unpublished data) by using IF/IR primer pair while the vector (pNWPH) backbone was linearized/amplified using VF/VR primer pair.

PCR reactions were performed in a mixture containing codon optimized GCSF gene as template, 1x HF buffer, 0.2 mM dNTPs, 0.5 *μ*M of each forward and reverse primer, and 5 units of* Pfu* DNA polymerase. The conditions used for high-fidelity PCR used for amplification are 98°C denaturation, 1 minute; 30 cycles of 98°C denaturation, 10 s; 64°C annealing, 20 s; and 72°C extension, 75 s, followed by 72°C extension for 5 minutes. The multimerization process of purified PCR products of the linearized vector and* GCSF* was performed through prolongeded overlap extension PCR essentially as described by You et al. [23] using high-fidelity* Pfu* DNA polymerase (0.04 U), dNTPs (0.2 mM for each), PCR-GCSF (2 ng/*μ*L), and PCR-linearized pNWPH (2 ng/*μ*L). The cycling profile was initial 98°C denaturation (30 sec.) and then 20 cycles of 98°C denaturation (10 sec.), 58°C annealing (30 sec.), and 72°C extension (3 min) followed by 15 cycles of 98°C denaturation (10 seconds) and 72°C annealing and extension (6 min) with final 72°C extension for 10 min (Figure 1).

*B. subtilis* SCK6 supercompetent cells were prepared essentially as described by X.-Z. Zhang and Y.-H. P. Zhang [21]. Briefly, LB medium (5 mL) containing 1 *μ*g/mL erythromycin was inoculated with the* B. subtilis* SCK6 and grown overnight at 37°C with constant shaking at 200 rpm. The overnight culture was diluted with fresh LB medium containing 2% (w/v) xylose to A_600_ of 1.0 and grown for another two hours.* B. subtilis* SCK6 strain contains additional copy of the* comK* gene, inserted downstream of the xylose promoter. Xylose, when added during the exponential phase of the SCK6 cells, acts as an inducer of the* comK* gene expression which adds up to the competency of cells. The resultant supercompetent cells were either used directly for the transformation or stored at −80°C as 10% (v/v) glycerol stocks.

For transformation, plasmid multimers (1 *μ*L) were mixed with 100 *μ*L supercompetent cells and incubated at 37°C for 90 min with constant shaking at 200 rpm. The positive transformants were selected on LB agar plates containing 5 *μ*g/mL chloramphenicol following incubation at 37°C for 14 hours. Modified alkaline lysis method [24], involving the treatment of cell pellet with lysozyme to break up the cell wall, was used to isolate the plasmid from two well-isolated positive colonies. Restriction digestion with* HindIII* and* NdeI* restriction endonucleases was performed to confirm the presence of insert in the isolated plasmids.

### 2.3. Expression in* Bacillus subtilis*

Transformed* B. subtilis* SCK6 cells, containing the recombinant human* GCSF*, were grown in two different media, LB and 2x L-Mal, at 37°C at 200 rpm in baffled Erlenmeyer flasks. For secretory expression, the cells were grown at low temperature, that is, 30°C, for a total of 120 hours. 1 mL sample aliquots were taken out at regular intervals of 12 hours until 120 hours and change in growth was monitored spectrophotometrically (OD_600_). Culture supernatant was examined for secretory expression of GCSF after centrifugation (6500 ×g, 4°C, 20 min) and precipitation through a modified TCA-acetone precipitation method. Briefly, to 1 mL of protein solution, 150 *μ*L TCA (100%) was added, placed at −20°C for 10 minutes, and then centrifuged at 14000 ×g for 5 minutes. Supernatant was discarded and the pellet was washed with 700 *μ*L of 100% ice-cold acetone to remove the residual TCA. The solution was placed at −20°C for 10 minutes prior to centrifugation. Second washing was done with 70% acetone and the pellet was dissolved in 50 mM Tris-Cl for use in subsequent analysis by 13% (w/v) SDS-polyacrylamide gel electrophoresis.

Bradford assay [25] and UV absorption method were used to measure the total secretory protein contents and purified recombinant GCSF concentration. Densitometric analyses of the SDS-gels were also used to determine the % of expression and/or the purity level of GCSF in different sample preparations.

### 2.4. Purification of Recombinant Human GCSF

For purification of rhGCSF, the culture supernatants of 72–80-hour fractions were subjected to salting out by ammonium sulphate precipitation. Ammonium sulphate was added slowly with constant stirring at 4°C to saturation of 65–80%. The precipitates were collected by centrifugation at 6500 ×g, 10 min, and dialyzed against 50 mM Tris-Cl (pH 8.5) buffer. The protein was subsequently purified on anion-exchange FPLC system, using 1 mL HiTrap QFF column (GE Healthcare). The column was preequilibrated with 50 mM Tris-Cl (pH 8.5). After sample injection, the column was washed with 2 column volumes of 50 mM Tris-Cl (pH 8.5) and the protein was eluted using linear gradient of 0 to 1 M NaCl in 50 mM Tris-Cl (pH 8.5).

### 2.5. Circular Dichroism Spectroscopy

Circular dichroism (CD) data of purified rhGCSF were collected on a ChirascanPlus CD spectrophotometer (Applied Photophysics, UK) equipped with a peltier thermal-controlled cuvette holder. For comparative purposes, CD spectra of the commercially available preparations of human GCSF (Filgrastim) were also obtained. Calibration was done with an aqueous solution of 1S-(+)-10-camphorsulfonic acid. The protein solution containing 156 *μ*g/mL in 10 mM Tris-Cl (pH 8.5) was scanned over wavelength 185 nm–260 nm at 2°C, using a quartz cell of 0.5 mm path length. Each wavelength spectrum was the result of averaging of two consecutive scans with a bandwidth of 1.0 nm. The wavelength spectra were refined by subtracting a blank spectrum with buffer only. The secondary structure content of protein was calculated using the CD spectrum deconvolution software CDNN [26] which calculates the secondary structure of the peptide by comparison with a CD database of known protein structures.

### 2.6. Biological Activity Assessment

Male mice each weighing 20–24 g were divided into two sets of 3 groups, each group consisting of four animals. They were fed ad libitum and maintained under controlled conditions of temperature (24–28°C), relative humidity (~65%), and artificial illumination (12 h per day). One set of three groups was used for administration of the drug. One of the groups was given in-house prepared rhGCSF, second group was given commercially available GCSF (Filgrastim, Sigma, USA), and the third group was given 0.1% BSA in 1x PBS (pH 7.4). The second set of three groups was treated in the same way except that the drug was administered through intraperitoneal route.

All the animals were given a single dose of ifosfamine (4.3 mg/0.5 mL) either through subcutaneous or intraperitoneal route to each animal of respective group to introduce neutropenia. Both the in-house produced rhGCSF and the commercial preparation were diluted to the concentrations of 15 and 40 *μ*g/mL in 1x PBS (pH 7.4) containing 0.1% BSA. The drug injections (1-2 *μ*g per gram of mouse weight) were administered one day after the injection of ifosfamine and continued daily for the next four days. Six hours after the last dose, peripheral blood samples were collected from orbital venous sinus. Glass slide smears were stained with May-Grunwald-Giemsa (Sigma) and the total number of neutrophils as well as the white blood cells was counted using a blood cell counter.

The percentage of neutrophils was calculated by taking mean ± SD of four animals for both routes of administration. By using GraphPad Prism Program (Version 4.0), one-way analysis of variance (ANOVA) followed by Bonferroni's posttest was performed to check the statistical significance of the data; *P* values were considered as significant when ≤ 0.05.

## 3. Results

### 3.1. Secretory Expression of rhGCSF in* B. subtilis*

The strategy for producing the pNWPH-GCSF vector, used for the secretory expression of* GCSF *in* B. subtilis*, is described in Figure 1. As shown, the codon optimized gene of GCSF is placed under the regulation of a strong P_HbaII_ promoter and the YwmC signal peptide encoding sequence (SPywmC) of* B. subtilis*. Nucleotides (~25) present at 5′ and 3′ termini of the insert and the vector, generated during PCR amplification, served as primers for each other and led to the formation of dimers during the first round of multimeric PCR. The dimers increased in number with each round of PCR cycle and finally the multimers were formed with repeated insert-vector-insert-vector sequences. The multimeric cloning strategy, used in the present study, involved the direct transformation of* B. subtilis* SCK6 supercompetent cells with the plasmid multimers, which is unlike the conventional cloning approach that includes additional steps of restriction digestion and ligation, prior to the transformation step.

**Figure 2:**
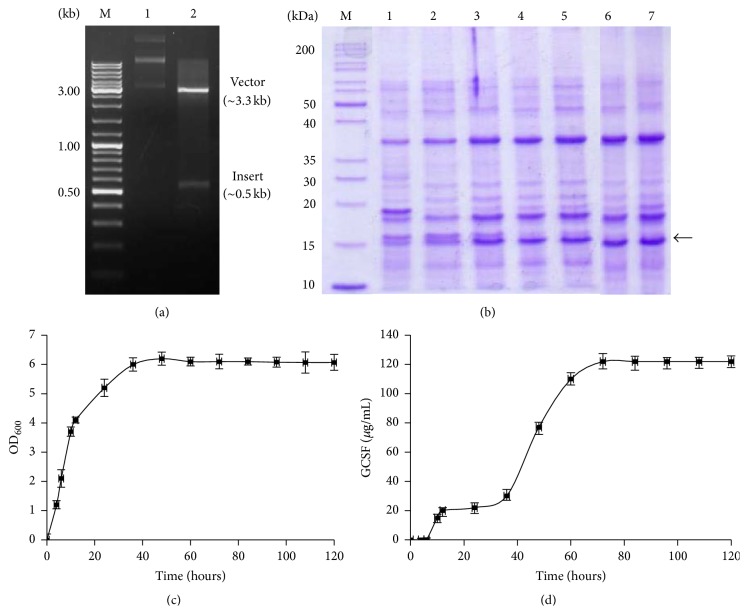
(a) Restriction analysis of pNWPH-GCSF expression plasmid resolved on 1% agarose gel. M, 1 kb DNA size marker; Lane 1, undigested pNWPH-GCSF; Lane 2, pNWPH-GCSF after double digestion with* NdeI* and* HindIII* restriction endonucleases. (b) 13% SDS-PAGE analysis of TCA-acetone precipitated culture supernatant of transformed* B. subtilis* SCK6. Lane M represents protein size marker; Lanes 1–7, sample fractions collected at 24, 36, 48, 60, 72, 84, and 96 hours of cell growth. (c) Growth of recombinant* B. subtilis* harboring pNWPH-GCSF in 2x L-Mal medium. *x*-axis shows time in hours while primary *y*-axis reflects the concentration of GCSF (*μ*g/mL) in culture supernatant, and secondary *y*-axis shows cell growth, monitored by absorbance measurement at 600 nm.

Positive transformants were selected using chloramphenicol as selection antibiotic while the presence and in-frame cloning of* GCSF* in pNWPH vector were confirmed through restriction digestion. Two bands, that is, ~3.3 kb of pNWPH vector and the ~0.5 kb GCSF insert, could be seen on 1% agarose gel following digestion of the recombinant plasmid with* NdeI* and* HindIII* (Figure 2(a)). Transformed* B. subtilis* SCK6 cells were grown in 2x L-Mal medium for 120 hours. Cell growth (OD_600_) was recorded (Figure 2(c)) and the secretory expression of GCSF at different stages was monitored by analysis of the sample aliquots of culture supernatant (Figures 2(b) and 2(d)).

When analyzed by SDS-PAGE, the culture supernatant of transformed* B. subtilis *SCK6 displayed a prominent band of ~19 kDa at 60 hours of growth which increased gradually with the passage of time. Maximum expression level, corresponding to ~17% of the total secretory protein, was attained at 72 hours, which remained constant until 96 hours. Thereafter, a sharp decline in cell growth was observed with a resultant drop in the levels of recombinant protein in the culture supernatant (Figures 2(c) and 2(d)).

### 3.2. Purification of rhGCSF

Secretion of recombinant proteins into the extracellular medium facilitates early downstream processing. For purification of GCSF, the culture supernatant was clarified by centrifugation and precipitated with 65–80% ammonium sulphate saturation. While very little amount got precipitated at 65%, highest amount could be recovered at 80% ammonium sulphate saturation with purity level of 75% (Table 2).

**Figure 3:**
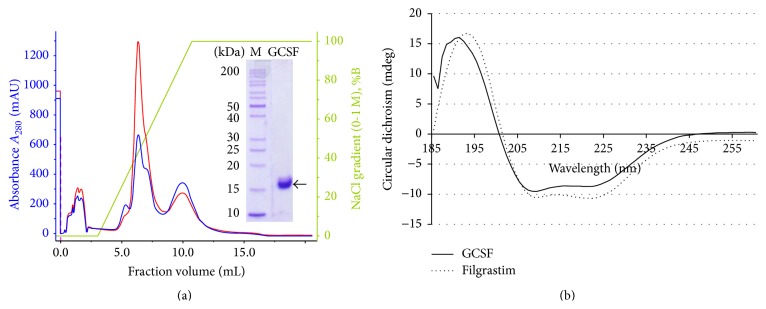
(a) Purification of recombinant human GCSF by FPLC on QFF column. Inset shows the purified GCSF eluted with 0.3 M NaCl concentration gradient. Blue and red colors show absorbance at A_280_ and A_260_, respectively. (b) CD spectrum of the recombinant in-house produced GCSF (solid line) and the commercially available GCSF preparation, that is, Filgrastim (dotted line), scanned over 185–260 nm range.

The collected fractions were dialyzed against 50 mM Tris-Cl to remove ammonium salt and the partially purified GCSF was purified to near homogeneity through anion exchange chromatography on FPLC as described in Section 2. The protein of interest eluted at ~0.3 M NaCl gradient, as shown in second peak of the chromatogram (Figure 3(a)). The GCSF purity level attained following two steps of purification was ~90% with a final recovery of 96 mg per liter of the culture supernatant (Table 2).

### 3.3. CD Spectrometry Analysis

CD spectrum of recombinant GCSF at 20°C showed double negative minima at 209 and 222 nm (Figure 3(b)). Analysis of the secondary structure using the CDNN software showed the presence of 57.8%  *α*-helices and 4.3% parallel and 4.2% antiparallel *β*-sheets. These secondary structure values are typical of a protein containing a large proportion of *α*-helical structure and are in coherence with the commercially available GCSF preparation. Since GCSF belongs to cytokine superfamily members containing *α*-helices and lack *β*-sheets, our data supports that recombinant GCSF produced in* B. subtilis* is in a properly folded conformation.

### 3.4. Biological Activity Assessment

**Figure 4:**
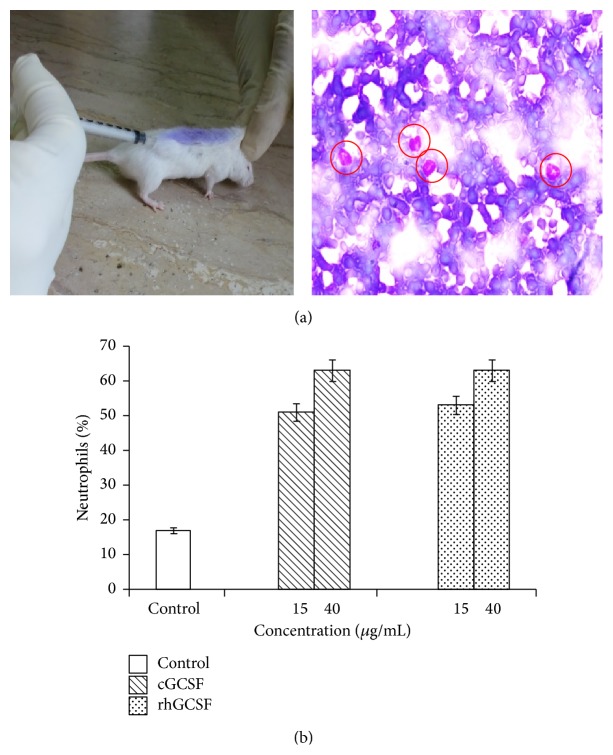
(a) GCSF biological activity assay. Left, mice being injected with GCSF by subcutaneous route; right, microscopic analysis of Giemsa stained slides wherein the encircled cells represent the neutrophil counts. (b) Mice in the sample and the control group received two different doses of GCSF (15 and 40 *μ*g/mL/mouse). The control group was treated with 0.1% BSA in PBS. The abbreviations cGCSF and rhGCSF stand for commercially available GCSF and in-house produced recombinant human GCSF, respectively.

The biological activity of recombinant, in-house produced GCSF was assessed in an* in vivo* model of neutropenia. Mice, treated with single dose of ifosfamine to induce neutropenia, were given recombinant GCSF and the percentage of neutrophils was monitored (Figures 4(a) and 4(b)). Amongst the two routes of drug administration tested in this study, that is, intraperitoneal and subcutaneous, the former delivery route of biosimilar was found to be more effective than the latter route (data not shown).

Statistically significant, dose-dependent increase in neutrophil count (*P* value < 0.001) was observed in the mice group treated with in-house produced GCSF. The trend was similar to what we observed in the group treated with commercially available Filgrastim (*P* value < 0.001). At 15 *μ*g/mL GCSF concentration, the increase in neutrophil count was up to 50% but improved further to a level of 60% with an increase in GCSF injection dose to 40 *μ*g/mL (Figure 4(b)). Overall, the effect of in-house produced GCSF and the commercially available filgrastim preparation on the two treated mice groups was statistically indistinguishable.

## 4. Discussion

Chemotherapy, in addition to killing cancer cells, often damages the rapidly dividing normal cells including the leukocyte producing bone marrow cells. Since leukocytes, more specifically neutrophils, play central role in defense against invading microbes, their reduced levels in response to chemotherapy or as a result of bone marrow transplantation make the body more susceptible to various life-threatening infections and sepsis [15, 27]. The injections of GCSF, either glycosylated or nonglycosylated, are therefore recommended and have been approved by US FDA for the treatment of chemotherapy-induced neutropenia, neutropenia caused by bone marrow transplantation, and neutropenia associated with the mylodysplatic syndrome or aplastic anemia [28]. Besides its applications in the treatment of neutropenia, GCSF has been found to have role in the treatment of central nervous system disorders like cerebral ischemia and strokes, regeneration of hepatic tissues, and so forth [16–18]. Therefore, biopharmaceutical companies, following the expiration of recombinant first-generation GCSF, are working on the production of new, bioactive GCSF biosimilars.

We, in the present study, were able to produce native-like, biologically active form of human GCSF in the culture medium by using a combination of pNWPH-GCSF expression vector and* B. subtilis* SCK6 host system. Multimeric cloning approach, which involves the use of POE-PCR, was opted for the construction of expression of plasmid pNWPH-GCSF (containing ~0.5 kb* GCSF* gene downstream of the P_HbaII_ promoter). This technique, originally described by You et al. [23], is relatively new but is simple and cost-effective and has certain advantages over the conventional cloning strategies, in particular the direct transformation of host without additional steps of restriction digestion and DNA ligation [22].

Amongst the commonly available expression hosts for the recombinant production of therapeutic proteins, namely, Chinese Hamster Ovary (CHO) cells, Human Embryonic Kidney (HEK) 293 cells,* Pichia pastoris* [29–32], and* E. coli*, the latter has widely been used to produce GCSF with high yields of up to 15 mg/L in shake-flask cultures [14, 33, 34]. Of note, the expression of GCSF in* E. coli*, reported in almost all the studies, was in the form of IBs, which demands use of denaturant (strong or mild) for solubilization and then removal of the denaturant as a prerequisite of refolding scheme [31, 32].

Earlier, we cloned and expressed the* GCSF* in* E. coli* BL21 (DE3) cytoplasm at levels corresponding to ~35% of total* E. coli* cellular proteins but in the form of IBs. The approaches used to improve the solubility of GCSF in* E. coli* transformants, that is, growth of transformed cells at low temperature (16–25°C), targeting of GCSF into the* E. coli* periplasm by attaching pelB leader sequence of the pET system, and the coexpression of* GCSF* with* M. tuberculosis* heat shock protein (Hspx), met with only limited success (unpublished results). However, use of* B. subtilis* as expression host in the present study resulted in enhanced and secretory expression of human GCSF with almost 6-fold higher yields than reported previously ([33] and references therein).

SPywmC, one of the powerful Sec-type peptides of the* B. subtilis* general secretory pathway (Sec pathway), was used for secretory expression of GCSF as used for heterologous expression of esterase previously [35]. When grown in 2x-LMAL nutrient enriched model medium [36–38], the cell growth increased gradually until the 50 hours and reached plateau afterward. The GCSF secretion, however, reached to maximum level (17%) at 72 hours, that is, during the stationary phase of cell growth (Figures 2(c) and 2(d)). These results are in good agreement with the nonclassical secretion of recombinant proteins in* B. subtilis* as reported previously [39]. Secretory expression facilitated rhGCSF downstream processing. By using ammonium sulphate precipitation and single FPLC column chromatography, >90% purity levels of recombinant protein were achieved. Purified GCSF injected in mice to assess its biological activity showed similar effect as commercially available Filgrastim, without any side effects on mice. Commercially available Filgrastim preparations were used to confirm the secondary structure of rhGCSF by circular dichroism. High *α*-helical content showed typical characteristic of cytokines [40]. In conclusion, this study reports for the first time the secretory expression of biologically active rhGCSF in* B. subtilis* SCK6 strain with minimum downstream processing steps and much higher yield than reported previously using the* E. coli* based expression system [33].

## 5. Conclusion

In conclusion, this study reports for the first time the secretory expression of biologically active rhGCSF in* B. subtilis* SCK6 strain with minimum downstream processing steps and much higher yield than reported previously using the* E. coli* based expression system. Our results showed that* B. subtilis* SCK6, with twofold advantages of convenient downstream processing and cost-effective high yield production of heterologous proteins (no inducer is required), may be exploited as an alternate expression system for the production of GCSF biosimilars.

## Figures and Tables

**Table 1:** Sequence of oligonucleotides used to amplify insert (IF/IR) and vector (VF/VR) DNAs during prolonged overlap extension (POE) PCR^*∗*^.

Primer	Sequence 5′-3′
VF	CCTTGCCCAGCCCTGATAGAAGCTTGGATCCGGAGTCGAACCATAAAAGC
VR	TGGCAGGGCCCAGGGGGGTCATATGAGCTGATGCCGAATACGTAAAGGTA
IF	TACCTTTACGTATTCGGCATCAGCTCATATGACACCTCTGGGCCCTGCCA
IR	GCTTTTATGGTTCGACTCCGGATCCAAGCTTCTATCAGGGCTGGGCAAGG

^**∗**^Primers were designed using online available software (http://www.xiaozhouzhang.com). AAGCTT and CATATG (underlined sequences) are the recognition sites for the *HindIII* and *NdeI* restriction endonucleases, respectively.

**Table 2:** Purification and recovery of human GCSF expressed in *B*. *subtilis*. Culture supernatant of transformed cells, grown in 1 liter of 2x L-MAL medium for 72 hours at 30°C with OD_600_ 6.0, was clarified by centrifugation and used for the purification of recombinant GCSF.

Steps	TSP^*∗*^	GCSF	Recovery	Purity
	(mg)	(mg)	(%)	(%)
Culture supernatant	720	122	100	17
Ammonium sulphate precipitation	235	115	94	49
Dialysis	212	110	90	52
FPLC purification (QFF)	107	96	78	90

^*∗*^TSP: total secretory protein.
